# Impact of acidic beverages on composition and surface characteristics of human teeth: scanning electron microscopic, stereomicroscopic and energy dispersive x-ray analyses

**DOI:** 10.1186/s12903-024-04491-4

**Published:** 2024-07-24

**Authors:** Naresh Kumar, Faiza Amin, Waheed Murad Dahri, Sara Khan, Huma Zaidi, Sehrish Rahman, Tooba Farhan, Muhammad Sohail Zafar, Muhammad Amber Fareed

**Affiliations:** 1 Department of Science of Dental Materials, Dr. Ishrat Ul Ebad Khan Institute of Oral Health Sciences, Dow University of Health Sciences, Karachi, 74200 Pakistan; 2 Department of Science of Dental Materials, Dow Dental College, Dow University of Health Sciences, Karachi, 74200 Pakistan; 3 Department of Science of Dental Materials, Bibi Aseefa Dental College, Shaheed Mohtarama Benazir Bhutto Medical University, Larkana, 77150 Pakistan; 4 Department of Restorative Dentistry, College of Dentistry, Taibah University, Al Madinah, Al Munawwarah, 41311 Saudi Arabia; 5 Centre of Medical and Bio-allied Health Sciences Research, Ajman University, Ajman, 346 United Arab Emirates; 6 School of Dentistry, University of Jordan, Amman, 11942 Jordan; 7 Department of Dental Materials, Islamic International Dental College, Riphah International University, Islamabad, 44000 Pakistan; 8 Clinical Sciences Department, College of Dentistry Ajman University, Ajman, 346 United Arab Emirates

**Keywords:** Acidic beverages, Demineralization, Erosion, Surface topography, Tooth composition

## Abstract

**Objectives:**

The objective of this study was to evaluate the effect of acidic beverages on the surface topography and elemental composition of human teeth.

**Methods:**

A total of five highly acidic beverages (Red Bull, Pepsi, Apple Cidra, Tang Mosambi, and Tang Orange) were investigated. The tooth specimens of experimental groups were submerged in each beverage and incubated at 37 °C for 7 days, whereas, the tooth specimens of control groups were placed in distilled water. Afterwards, tooth specimens were analyzed using scanning electron microscopic (SEM), stereomicroscopic, and energy dispersive x-ray (EDX) techniques.

**Results:**

All experimental groups revealed a decline in the tooth elements compared to controls, however, such decline was not statistically significant. Nevertheless, comparing the experimental groups, the Red Bull beverage caused a marked reduction in the percentage of both calcium and phosphorus elements compared to the Pepsi, Apple Cidra, Tang Mosambi, and Tang Orange beverages but it was insignificant as well in contrast to its control counterpart. All five acidic beverages demonstrated erosive potential under SEM analysis; however, each group of specimens showed a diverse amount of demineralization. In addition, all experimental groups exhibited significant discoloration of tooth specimens compared to their respective control counterparts.

**Conclusions:**

Within the limitations of study, all five acidic beverages demonstrated erosive potential in the simulated in vitro conditions under SEM analysis; however, each group of specimens exhibited a different extent of demineralization. In addition, the overall effect of all beverages was insignificant under EDX analysis as no substantial difference was revealed between the elemental composition of experimental and control group specimens.

## Introduction

The incidence and prevalence of dental erosion are on the sharp rise worldwide [1]. Dental erosion (also known as tooth erosion) is defined as non-carious chemical loss of dental tissues in the absence of bacteria [2]. According to the origin of etiological acids, tooth erosion can be classified into intrinsic or extrinsic [3]. The main cause of intrinsic tooth erosion is the introduction of gastric acids into the oral cavity, which includes pathological medical conditions such as gastroesophageal reflux disease (GERD), bulimia nervosa, chronic alcoholism, and hyperemesis gravidarum. Extrinsic factors include a combination of dietary acids present in beverages, certain medications, lifestyle, and environmental or occupational factors (wine tasters, manufacturing electrolytic/galvanic batteries, etc.) [4–6]. Initial softening (hardness loss) of the surface is observed in enamel erosion which is followed by permanent loss of tooth volume because of the dissolution of enamel crystals. After the initial acid attack, the surface layer of the remaining enamel becomes remarkably softer [7, 8]. Theoretically, it is postulated that remineralization and repair are still possible at the early stage of the enamel erosion process because the residual enamel may act as a scaffold and support the repair of the damaged tissues. However, repair of the enamel tissue is not possible as the outer layer of the enamel is totally devoid of minerals in the advanced stage of the erosion process [9]. Possible effects of dental erosion include pain, dentine hypersensitivity, compromised esthetics, temporomandibular dysfunctions, and reduced oral functions [10]. To prevent dental erosion, various self-care measures have been suggested by dental professionals in addition to the biomimetic restorative and endodontic approaches [6, 11, 12].

Incredibly, striving for a healthier and better lifestyle can inappropriately result in dental health issues such as dental erosion. Regular exercise and diets rich in acidic food and beverages are the main factors that provoke the lifestyle change. Intake of carbonated and uncarbonated/acidic beverages, sports drinks, and fruit juices is likely to be increased during exercise because of decreased salivary flow. It has been observed that the severity of gastroesophageal reflux is increased during intense workouts [13]. It has been hypothesized that a vegetarian diet and excessive use of vinegar dressings could potentially contribute to dental erosion. However, the critical analysis of this topic indicated that there are significant limitations regarding the evidence of erosivity of these vegetarian diets and vinegar dressings [14, 15]. Airborne industrial acids have been implicated in dental erosion among factory workers, particularly in munitions, battery, and fertilizer plants. People who regularly swim in chlorinated swimming pools were exposed to the high acidic pH of the pool water and develop dental erosion more frequently than the non-swimmers [16].

According to the global prevalence, the dental erosion of permanent and primary dentitions ranges from 20 to 45% and 30–50%, respectively [1]. However, it is very challenging to estimate the true global prevalence of dental erosion due to differences in sample size, indices and study design [1]. According to a meta-analysis, 34.1% of worldwide children and adolescents suffered from dental erosion worldwide [17] . According to the National Health and Nutrition Examination Survey (NHANES, 2003–2004) conducted in the U.S. found that 80% of adults and 45.9% of children suffer from erosive tooth wear [18]. Recently in 2022, another survey has been conducted by an Australian university aiming to measure awareness and knowledge of dental erosion among undergraduate students and to develop the relationships between dental erosion with sociodemographic factors and beverage consumption behaviors [19].

This study is the continuation of our recently published work [20], in which we investigated the pH of one hundred and six beverages commonly used by the public. Surface hardness and weight loss were evaluated to investigate the effects of these beverages on human tooth specimens. We found that the pH levels of commonly available beverages including, Apple Cidra™, Pepsi™, Red Bull™, Tang Mosambi™ as well as Tang Orange™ highly acidic, which might lead to minerals loss from teeth and reduced surface hardness of the specimens compared to the control. Moreover, after evaluating the surface hardness, weight loss and having remarkable findings, this study aimed to evaluate the effects of these five most acidic drinks on tooth structure to identify the effect of these drinks on the surface topography and elemental composition by the scanning electron microscope (SEM), stereomicroscope and energy dispersive X-ray (EDX) techniques.

## Materials and methods

This study is a continuation of our previous research [20] where we measured the pH levels of 106 beverages available in local supermarkets in Karachi, Pakistan. Based on the results, we identified five highly acidic beverages, namely Red Bull™ (Karachi, Pakistan), Pepsi ^TM^ (Karachi, Pakistan), Apple Cidra™ (Karachi, Pakistan), Tang Mosambi™ (Karachi, Pakistan), and Tang Orange™ (Karachi, Pakistan) for further evaluation regarding their effects on tooth structure loss and surface hardness. In this study, we aimed to examine the effects of these drinks on the tooth enamel surface topography, discoloration and composition of tooth structure using scanning electron microscope (SEM) and stereomicroscope and energy dispersive X-ray analyses. Institutional review board (IRB) approval was obtained for this study (IRB-1648/DUHS/Approval/27 June 2020) at the Dow University of Health Sciences, Karachi, Pakistan.

### Drinks preparation

The powdered drinks, namely Tang Mosambi™ and Tang Orange™, were prepared in accordance with the instructions provided on the packaging. On the other hand, the ‘Ready to drink’ beverages, including Red Bull™, Pepsi™, and Apple Cidra™, were shaken properly before opening. The compositions of each beverage are illustrated in Table 1.

**Table 1:** The compositions of beverages used in this study

Beverage	Water (%)	Sugar (g/100 ml)	Citric Acid (g/100 ml)	Caffeine (mg/100 ml)	Other Ingredients
Red Bull™	91	11	0.34	32	Taurine, B-vitamins, Phosphoric acid etc.
Pepsi™	89	10.6	0.15	10	Caramel color, Phosphoric acid, etc.
Apple Cider™	88	10	0.4	Trace	Apple juice, Preservatives, etc.
Tang Mosambi™	94	8	0.2	None	Mosambi flavor, Artificial colors, etc.
Tang Orange™	93	9	0.3	None	Orange flavor, Artificial colors, etc.

### Sample preparation

**Fig. 1:**
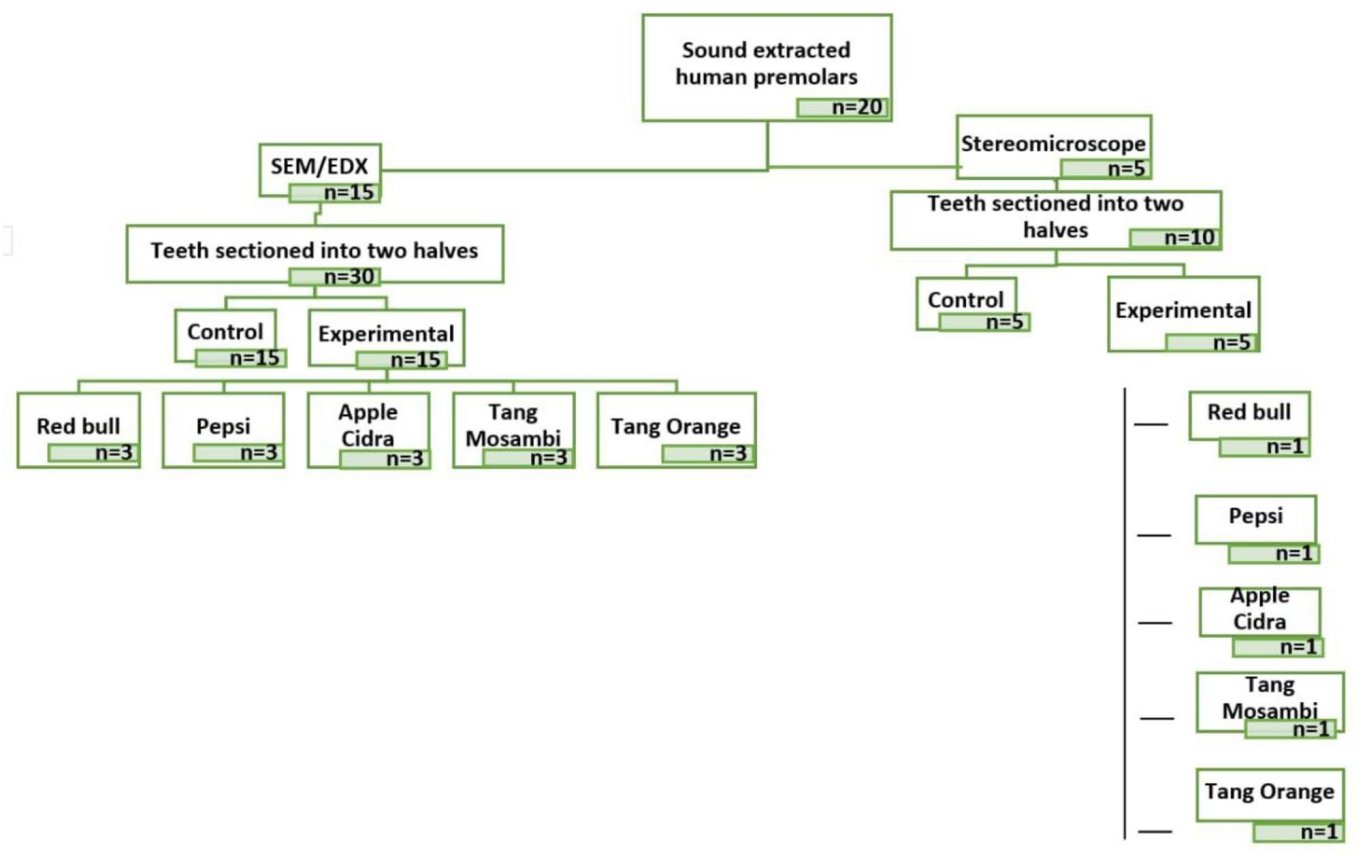
Flow chart illustrating sample size distribution

A total of 20 healthy premolars extracted for orthodontic purposes were obtained from the Department of Oral Surgery, Dow University of Health Sciences (DUHS), Karachi, Pakistan, Prior to use, informed consent was obtained from all patients. The teeth were carefully cleaned and disinfected using a 0.5% chloramine T trihydrate solution and scaled using a dental scaler (Master 400; EMS Peizon, Nyon, Switzerland) to remove any calculus and debris and to ensure that any pathological conditions were excluded. Each tooth was then longitudinally sectioned into two parts using diamond cutting disc (MANI devices and instruments; Takenzawa, Japan) in a Micromotor (K-35 Cube 40,000 rpm; Seyang Micro Tech Co, Daegu, Korea) and the sectioned parts of each tooth were stored in a separate container containing distilled water until further experimentation [19]. Before testing, each tooth section was manually polished with progressively finer silicon carbide papers with grit sizes of 600, 1200, 2500, and 4000 to remove any debris, plaque, or foreign particles [20]. The specimens were approximately 3 mm thick. To assess the effects of five highly acidic beverages (Red Bull™, Pepsi™, Apple Cidra™, Tang Mosambi™, and Tang Orange™) on the tooth enamel surface, structure and composition, each beverage was prepared in separate containers, and then 20 sectioned parts of twenty teeth were submerged in the beverages (Red Bull; *n* = 4), Pepsi; *n* = 4, Apple Cidra; *n* = 4, Tang Mosambi; *n* = 4, and Tang Orange; *n* = 4) at 37 °C for 7 days [20]. The beverages were replaced every day until the 7-day period was completed. Whereas the remaining 20 sectioned counterparts of twenty teeth were left in the distilled water until evaluation which served as controls (Fig. 1).

**Fig. 2:**
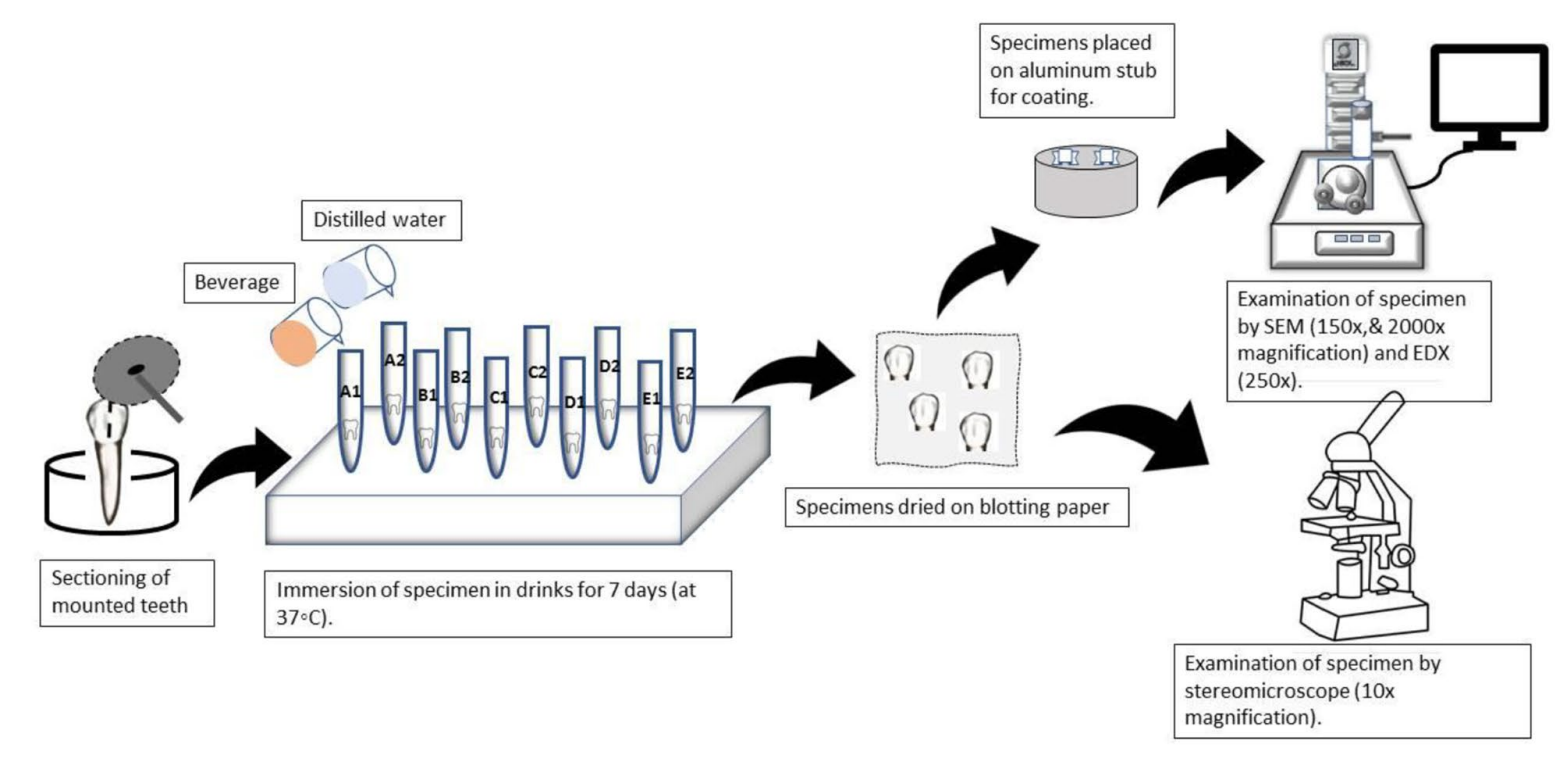
A schematic diagram showing a complete representation of the experiment from tooth sectioning to scanning electron microscopic, stereomicroscopic and energy dispersive x-ray analyses

The sectioned teeth specimens were then examined under a SEM (JSM-IT100; JEOL, USA) and a stereomicroscope (M205C Microscope; LEICA, Germany) to evaluate any changes in tooth enamel surface and structure caused by the acidic beverages. In addition, EDX (JSM-IT100; JEOL, USA) was also done to evaluate the changes in the mineral content of the tooth caused by these beverages (Fig. 2).

### SEM and EDX analysis

After the completion of the storage period, the specimens from experimental groups (*n* = 3 per group) were washed with distilled water and dried using blotting paper before SEM and EDX analysis [21]. The samples were coated with a thin layer of platinum using a sputter coater (Auto Fine Coater – JEC-3000FC; JEOL, USA). Covering samples with a thin layer of conductive material like Gold or platinum for SEM analysis is a widespread practice, particularly with non-conductive samples [21]. Platinum was used as it is cost effective. Although not universally required, it is often utilized to enhance imaging quality and reduce artifacts arising from charge accumulation. The SEM (JSM-IT100; JEOL, USA) was used to observe the specimens at a magnification of 150x and 2000x, with an accelerating voltage range of 500 V to 30 kV . Subsequently, EDX was performed on those coated samples, at a magnification of 250x. Similarly, SEM and EDX analyses of each control group specimens (*n* = 3 per group) were performed using the same magnification and voltage range as mentioned above.

### Stereomicroscopy

After the immersion period, one specimen from each experimental group was washed and dried, and their surface discoloration was analyzed using a stereomicroscope (M205C Microscope; LEICA, Germany) at 10x magnification. Likewise, one specimen from each control group was also analyzed using a stereomicroscope (M205C Microscope; LEICA, Germany) at 10x magnification and then the discoloration of specimens from both control and experimental groups was compared.

### Data analysis

Data entry and statistical analysis was carried out using the SPSS v21 software; IBM. Mean and Standard deviation were obtained. The student’s t-test was conducted on the EDX data of each beverage group to highlight the difference between means of control and experimental group specimens. The SEM and stereomicroscopic data of specimens were presented in images to highlight the surface topography and discoloration of both control and experimental group tooth specimens. *P*-value < 0.05 was considered significant.

## Results

Apparently, there was a decline in both calcium and phosphorus elements of the tooth after immersion in each beverage as compared to the control specimens, however, the student’s test-test did not reveal any significant difference between the means of control and experimental group specimens (*p* > 0.05) (Table 2).

**Table 2:** Mean at% and standard deviation of elemental calcium and phosphorus present in control and experimental groups’ tooth specimens

Beverage	Tooth Elements	Groups	Mean % (Standard Deviation)	Tooth elemental loss after immersion in a beverage (%)	*p* – value
Red Bull	Calcium	Control	30.22 (2.43)	49.24	0.09
		Experimental	15.34 (11.29)		
	Phosphorus	Control	16.74 (0.50)	50.06	0.12
		Experimental	8.36 (7.44)		
Pepsi	Calcium	Control	35.34 (2.42)	16.76	0.12
		Experimental	29.42 (4.79)		
	Phosphorus	Control	19.87 (1.72)	14.25	0.07
		Experimental	17.04 (1.11)		
Apple Cidra	Calcium	Control	38.31 (5.68)	22.53	0.37
		Experimental	29.68 (13.79)		
	Phosphorus	Control	20.62 (3.20)	16.30	0.44
		Experimental	17.26 (6.03)		
Tang Mosambi	Calcium	Control	30.06 (3.70)	5.19	0.57
		Experimental	28.50 (2.45)		
	Phosphorus	Control	18.98 (3.11)	10.22	0.34
		Experimental	17.06 (0.25)		
Tang Orange	Calcium	Control	31.70 (2.67)	32.09	0.42
		Experimental	21.53 (17.85)		
	Phosphorus	Control	17.54 (2.53)	28.00	0.38
		Experimental	12.63 (8.41)		

*P* > 0.05 indicates no significant difference between the means of control and experimental groups for each beverage

Among all study groups, the Red Bull beverage caused a marked reduction in the percentage of both calcium and phosphorus elements as compared to the Pepsi, Apple Cidra, Tang Mosambi and Tang Orange beverages (Table 2).

**Fig. 3:**
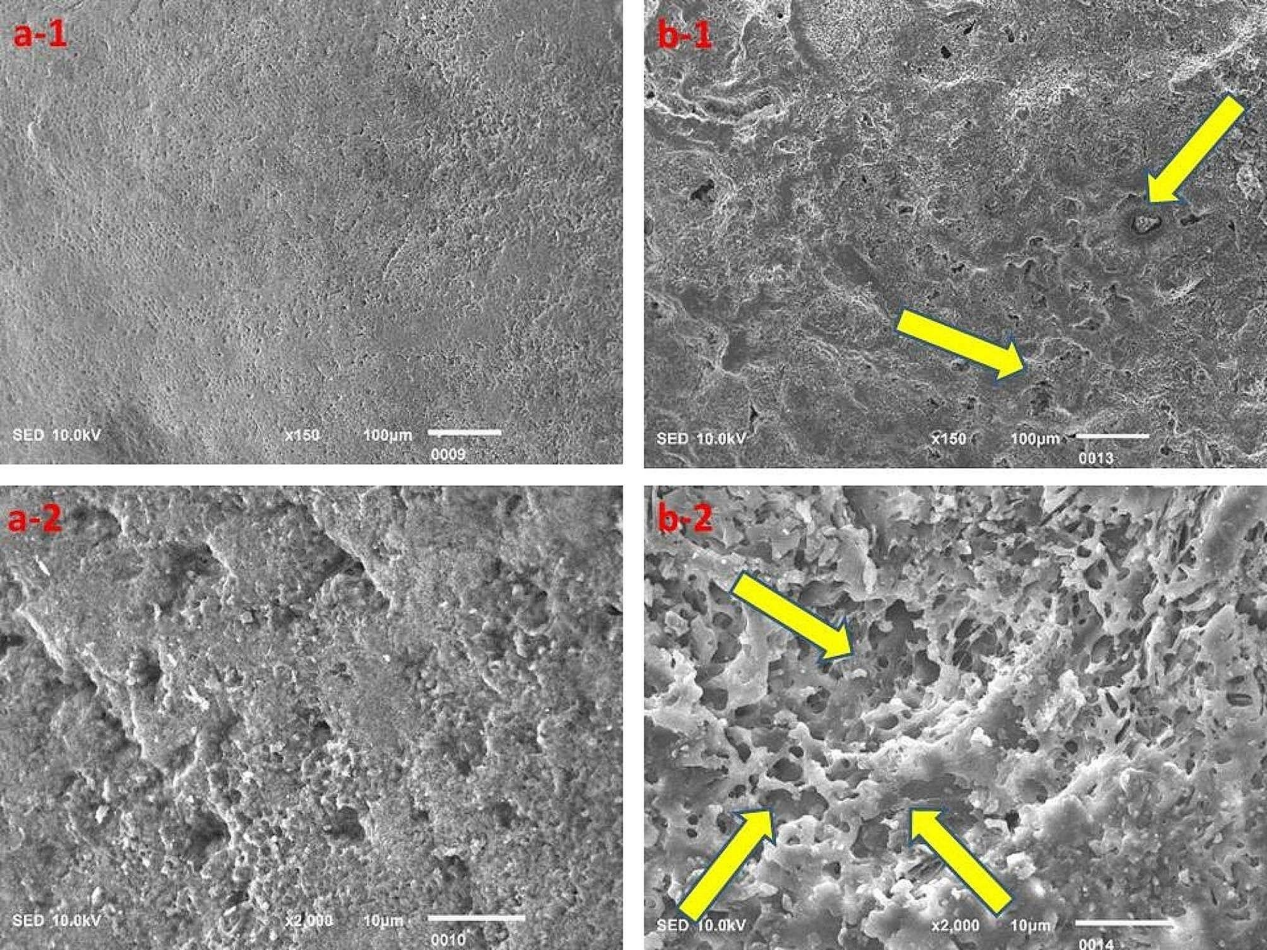
Surface topography of the control group (**a-1** and **a-2**) and experimental group (**b-1** and **b-2**) tooth specimens after storage in the Red Bull beverage. The highlighted areas (arrows) indicate a degree of demineralization of tooth specimens

**Fig. 4:**
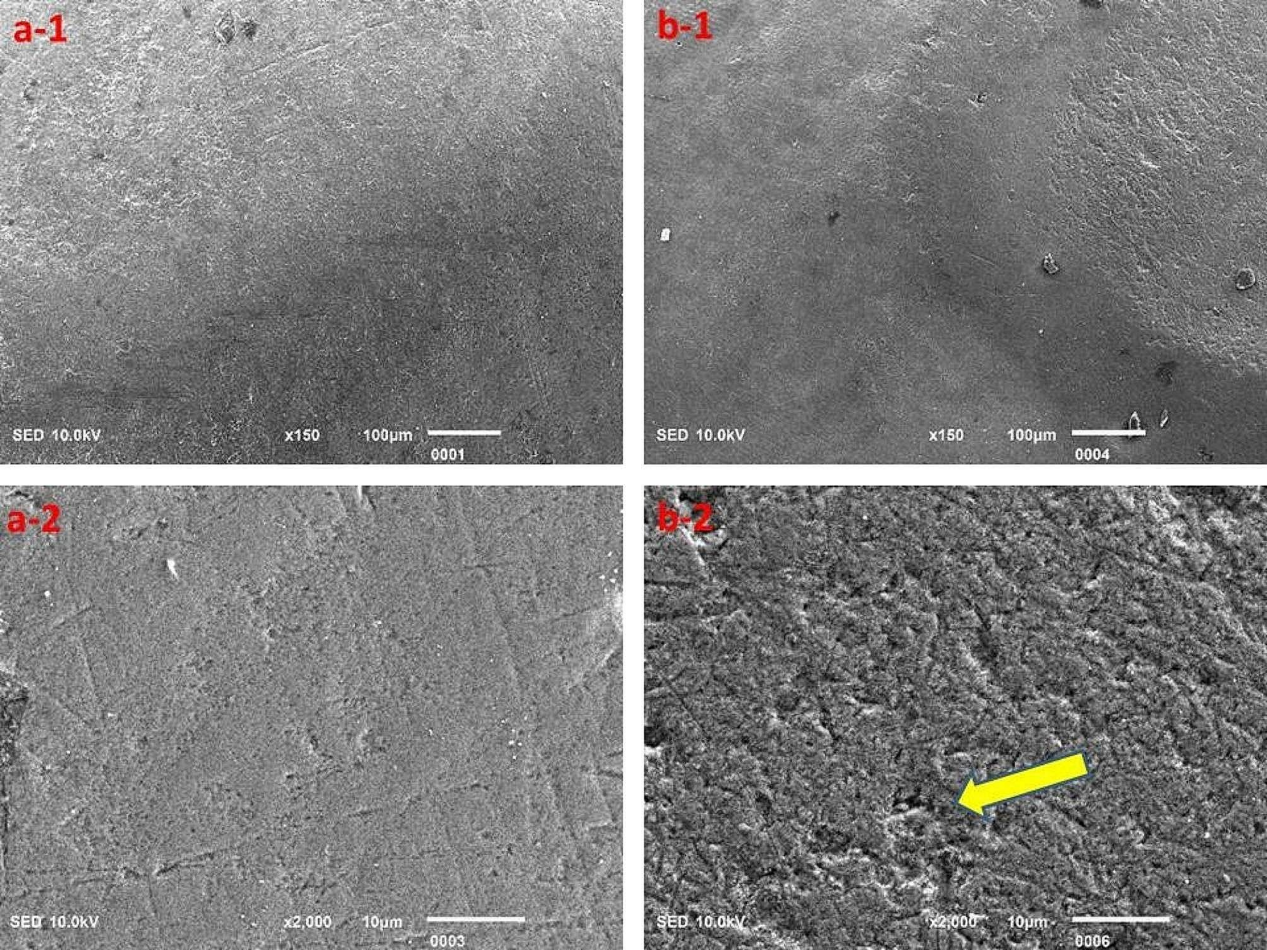
Surface topography of the control group (**a-1** and **a-2**) and experimental group (**b-1** and **b-2**) tooth specimens after storage in the Pepsi beverage. The highlighted area (arrow) indicates the degree of demineralization of the tooth specimen

**Fig. 5:**
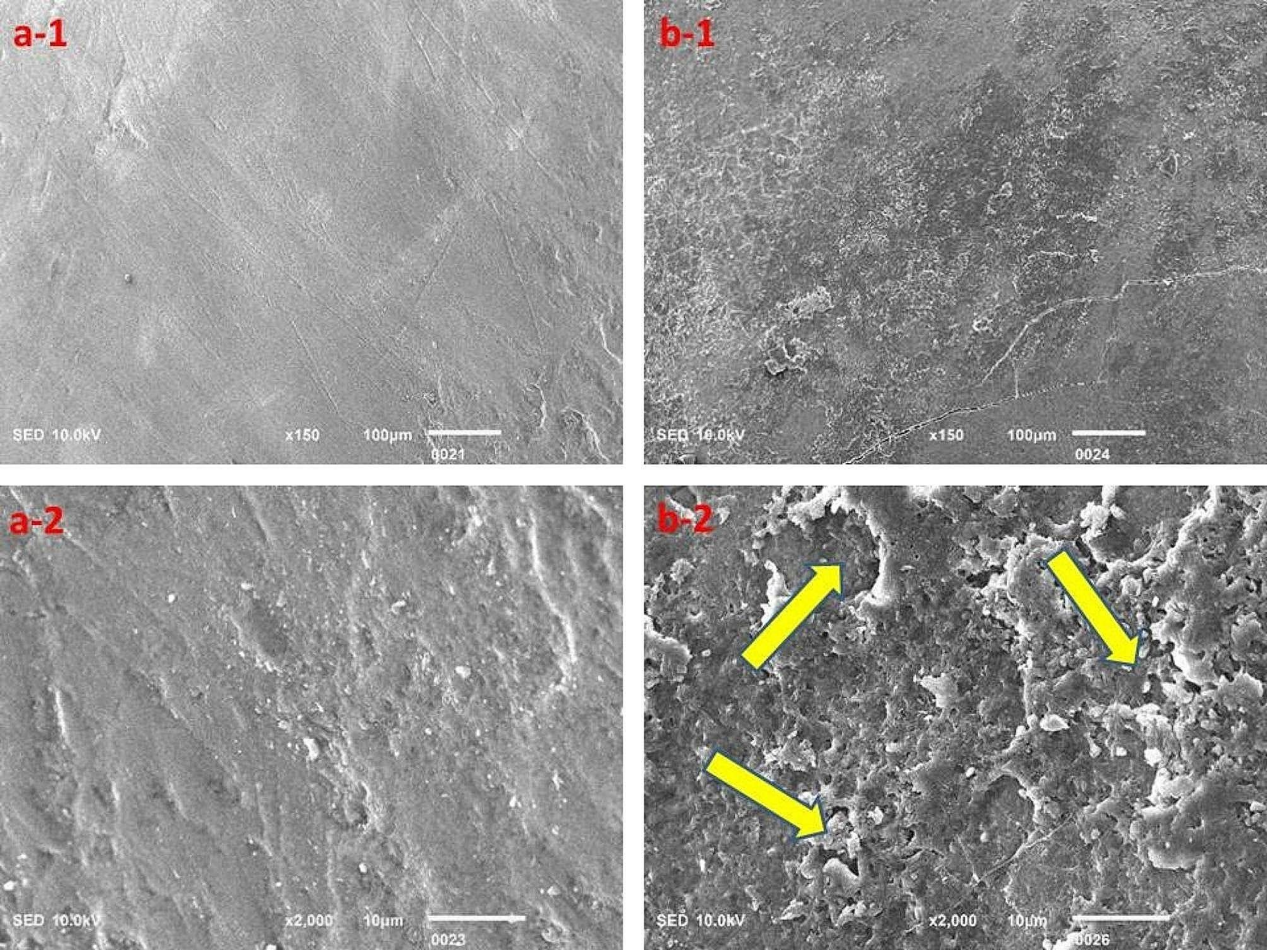
Surface topography of control group (**a-1** and **a-2**) and experimental group (**b-1** and **b-2**) tooth specimens after storage in the Apple Cidra beverage. The highlighted areas (arrows) indicate the degree of demineralization of tooth specimens

**Fig. 6:**
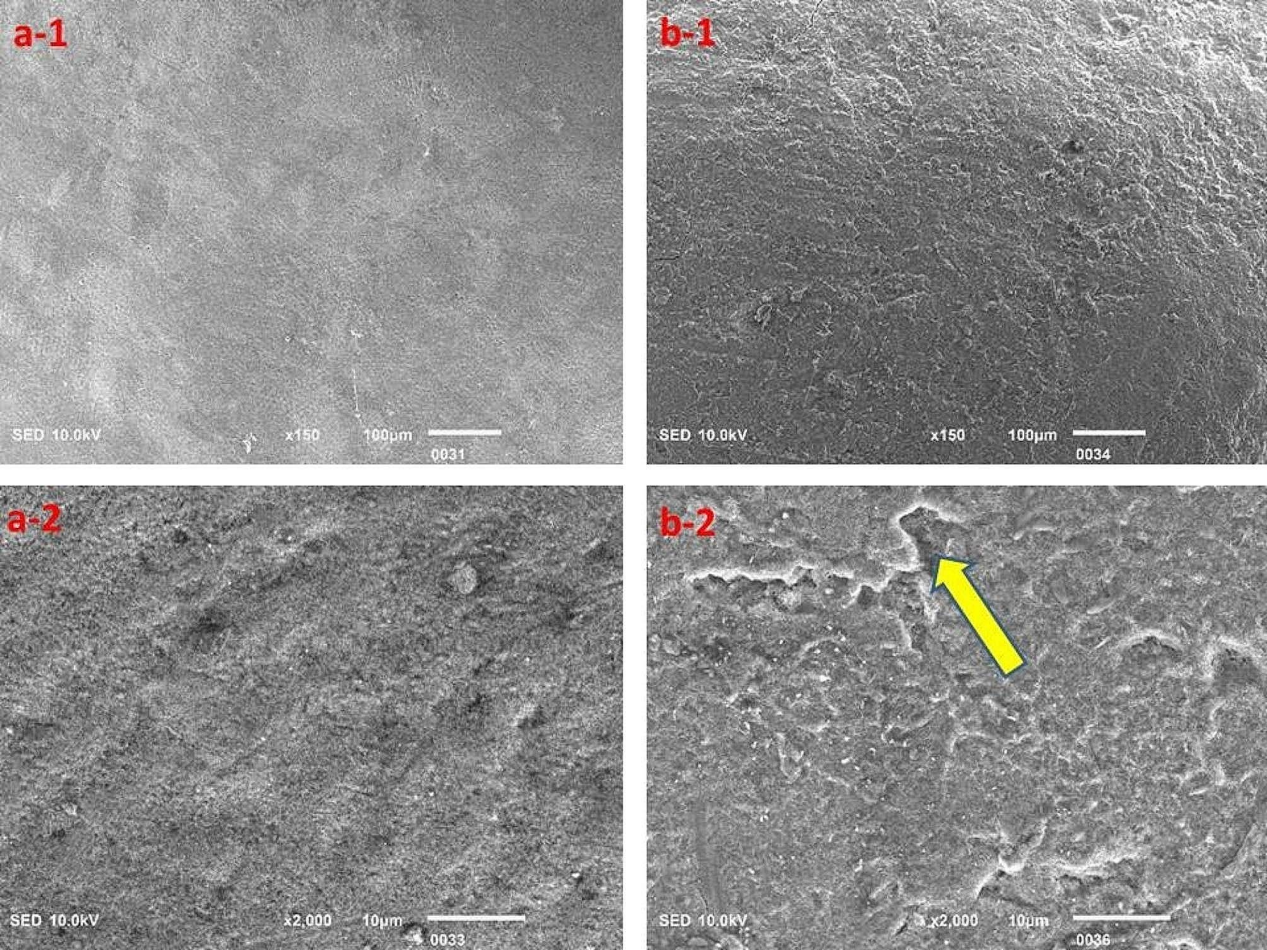
Surface topography of control group (**a-1** and **a-2**) and experimental group (**b-1** and **b-2**) tooth specimens after storage in the Tang Mosambi beverage. The highlighted area (arrows) indicates the degree of demineralization of the tooth specimen

**Fig. 7:**
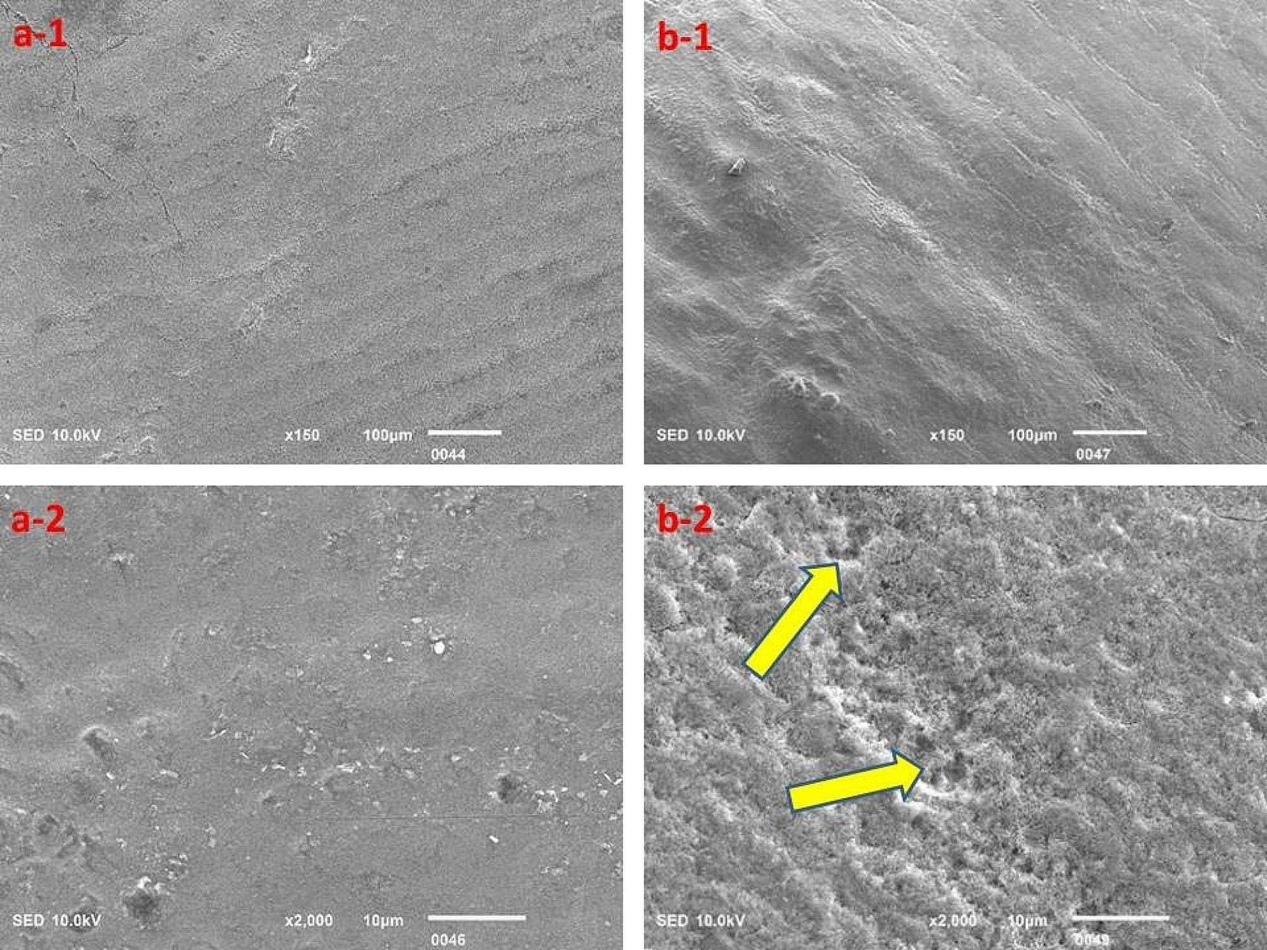
Surface topography of control group (**a-1** and **a-2**) and experimental group (**b-1** and **b-2**) tooth specimens after storage in the Tang Orange beverage. The highlighted areas (arrows) indicate the degree of demineralization of tooth specimens

The surface topography clearly highlighted the acidic effect; however, the Red Bull appeared to cause relatively greater destruction of tooth structure as compared to the rest of the beverages investigated in this study, but the results were insignificant (Figs. 3, 4, 5, 6 and 7).

**Fig. 8:**
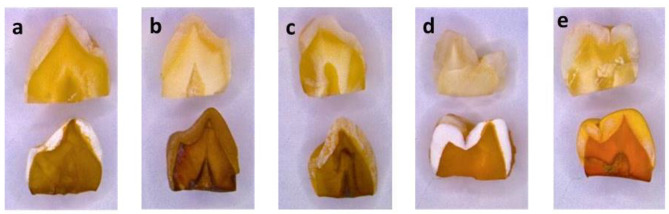
Stereomicroscopic analysis highlighting the discoloration of tooth specimens following immersion in **(a)** Red Bull, **(b)** Pepsi, **(c)** Apple Cidra, **(d)** Tang Mosambi and **(e)** Tang Orange beverages compared to their control counterparts

In addition, all the beverages caused significant discoloration of specimens as compared to their respective control counterparts (Fig. 8).

## Discussion

In the present study, an attempt was made to explore the influence of acidic beverages on human teeth using various characterization techniques including SEM, stereomicroscope and EDX. The variables namely tooth composition, surface topography and color were affected by all the acidic beverages in the current study which clearly support the findings of our previous study where we evaluated the impact of acidic beverages on human teeth using surface hardness and weight loss methods in our previous study and identified the significant effect of each beverage [20]. The sample size in in-vitro studies frequently validate adequate statistical power with sample sizes as small as three, thus we chose *n* = 3 for SEM analysis [21]. In the literature, various methods for instance scanning electron microscopy, atomic force microscopy, microradiography, surface hardness, weight loss, chemical analysis and digital image analysis have widely been utilized to assess tooth erosion [22]. While procedures like profilometry offer quantitative data, their integration would have expanded the study’s scope away from its intended center. To warrant clarity and coherence, we selected qualitative approach that directly lined up with our research objectives.

During EDX analysis, all beverages led to a decline in Ca and P content of experimental group specimens compared to the control group specimens, but the decline was insignificant in terms of p-value. This finding is in agreement with a previous study by Sooksompien et al. [23]. , which demonstrated a reduction in the Ca and P elements of tooth specimens following immersion in three acidic drinks. Likewise, Caneppele et al. [24]. , evaluated the mineral concentrations of bovine teeth specimens following immersion in five acidic beverages and found a substantial decrease in Ca and P elements following immersion in Red Bull beverage compared to its reference group. This finding agrees with our study as 49.24% and 50.06% loss of Ca and P respectively were observed after the immersion of specimens in the Red Bull beverage in contrast to the respective control group. The loss of minerals is observed, but no statistically significant difference was found between the control and experimental group. It might be due to the specific components of beverages. Components like phosphoric acid, citric acids, sugar, and caffeine might create an acidic environment, whereas water, the major component of beverages might be balancing the demineralization as water is neutral and does not hold erosive potential for tooth enamel. Furthermore, increasing the frequency, duration or temperature or other factors might cause a significant damage [25].

The loss of both mineral Ca and P in the Red Bull-treated specimens is greater as compared to the rest of the specimens immersed in other beverages, despite the insignificant difference in the pH values of all beverages. This outcome may be attributed to the varied composition of Red Bull. It is certainly understandable that the constituents of beverages play a vital role in dental erosion since the availability of sugars and citric acids in beverages has widely been linked to dental erosion as shown in Table 1. Further research work regarding the exact formulation of beverages is warranted in an attempt to explore the key factors responsible for such an outcome [26, 27]. There were marked differences in the Ca and P values of specimens among the study groups. Since it was practically impossible to include the teeth of a single patient, hence variability in the enamel rods, the direction of the crystallites within the rods, the extent of demineralization, and the existence of fluoride ions are anticipated. Consequently, this might have resulted in variations in the elemental composition among the specimens analyzed in the current study.

The SEM analysis revealed evident signs of demineralization of the tooth specimens that were immersed in different acidic beverages (Figs. 3, 4, 5, 6 and 7), like shallow depressions, irregular or uneven texture, cracks, irregular patterns or lines, and porosities due to dissolution of mineral components from tooth enamel. However, each group of specimens exhibited a different extent of demineralization which is in line with the EDX data of the current study. The variation in the degree of demineralization of each group of specimens can be attributed to the distinct composition of each beverage (Table 1). Among all groups, the specimens immersed in the Red Bull beverage demonstrated the greatest demineralization (Fig. 3), which might be due to the presence of phosphoric acid, citric acid along with the highest caffeine and sugar constituents among other beverages. It clearly supports the EDX data as about half of the Ca and P were lost after the immersion of specimens in the same beverage (Table 2). The SEM findings of our research work concur with the previous studies [23, 28, 29]. For instance, Jameel et al. [28]. , conducted the SEM analysis of teeth after exposing them to two acidic beverages, Coca-Cola and orange juice, and reported surface irregularities due to the dissolution of enamel rods and inter-rod area. In addition, differences in the demineralization pattern of both groups were identified. Grando et al. [29]. , also observed noticeable erosion of the tooth specimens after exposing them to lemon juice and Coca-Cola as the sheaths and heads of enamel prisms were severely damaged. Likewise, findings of a study by Sooksompien et al. [23]. , reveal erosion of the enamel surface after immersion in the acidic beverages by highlighting coral network and map-like appearance along with a heterogeneous pitting pattern.

The stereomicroscopic analysis of tooth specimens after immersion in the acidic beverages exhibited severe discoloration as compared to the control group specimens and these findings are in line with previous studies [29, 30]. Maladkar et al. [30]. , evaluated the effect of acidic beverages and dietary preservatives on extracted human teeth and they observed severe discoloration of teeth after their exposure to Coca-Cola, Red Bull, and Mountain Dew. Similarly, Grnado et al. [29]. , also observed the light-brown color of teeth while exposed to Coca-cola. This discoloration of specimens may be ascribed to the presence of coloring agents in these acidic beverages [30]. Erdemir et al. [31]. , have made the presence of caffeine, taurine, ginseng, and guarana extract responsible for the discoloration of teeth. In the current study, we did not observe any loss of gloss or surface roughness in the experimental group specimens using a stereomicroscope. On the contrary, the findings of a study by Maladkar et al. [30]. , revealed loss of gloss and roughness of tooth structure following exposure to acidic beverages. This difference may be attributed to the exposure time of specimens.

The composition of beverages shows a discrepancy from country to country owing to variances in sourcing of ingredients, regulations, and local preferences. Even though the principal constituents of Red Bull™, Pepsi™, Apple Cidra™, Tang Mosambi™, and Tang Orange™ may stay consistent across states, deviations in flavorings, preservatives, sweeteners, and supplementary additives may perhaps occur to cater to native tastes and regulatory requirements. Moreover, factors for instance sourcing of ingredients and industrial processes can also impact the composition of beverages in distinct countries. Hence, it is probable that the composition of selected beverages in Pakistan vary to some extent from that in other countries and this has not been evaluated before up to our knowledge. Thus, these beverages are consumed by children and pupil in Pakistan very commonly, considering harmless. This study spreads awareness that it should be consumed with precautions and not considered harmless to teeth as they do possess erosive potential.

This in vitro study has a few limitations. For instance, the flushing effect of saliva and its buffering capacity were not replicated, although these factors may affect the erosive potential of acidic beverages. In addition, alcoholic beverages were not considered in this study owing to religious and cultural restrictions.

## Conclusions

Within the limitations of study, all five acidic beverages demonstrated erosive potential in the simulated in vitro conditions under SEM analysis; however, each group of specimens exhibited a different extent of demineralization. In addition, the overall effect of all beverages was insignificant under EDX analysis as no substantial difference was revealed between the elemental composition of experimental and control group specimens.

## Data Availability

All data generated or analysed during this study are included in this published article.
